# Supplementary material for: Self-concept in poor readers: a systematic review and meta-analysis
Source: PeerJ. 2020 Mar 16;8:e8772. doi: 10.7717/peerj.8772 (PMC7081778; doi:10.7717/peerj.8772)
Supplement: Appendix S1 [file peerj-08-8772-s002.docx]

| **Appendix A**  Data Extraction Sheet | | | | |
| --- | --- | --- | --- | --- |
| **Descriptive Information** | | | | **Data** |
| Paper 1 | Author | | |  |
|  | Title | | |  |
|  | Year | | |  |
|  | Reading measure | Name | |  |
|  |  | Subscales | 1 |  |
|  |  |  | 2 |  |
|  |  |  | 3 |  |
|  | Self-concept measure | Name | |  |
|  |  | Subscales | 1 |  |
|  |  |  | 2 |  |
|  |  |  | 3 |  |
|  | Group 1 | Group Type | | Typical developers |
|  |  | N | |  |
|  |  | Reading score | M |  |
|  |  |  | SD |  |
|  |  | Self-concept score | M |  |
|  |  |  | SD |  |
|  | Group 2 | Group Type | | Poor readers |
|  |  | N | |  |
|  |  | Reading score | M |  |
|  |  |  | SD |  |
|  |  | Self-concept score | M |  |
|  |  |  | SD |  |
