# Supplementary material for: Self-concept in poor readers: a systematic review and meta-analysis
Source: PeerJ. 2020 Mar 16;8:e8772. doi: 10.7717/peerj.8772 (PMC7081778; doi:10.7717/peerj.8772)
Supplement: Appendix S2 [file peerj-08-8772-s003.docx]

| **Appendix X**  Correspondence with authors | | | | |
| --- | --- | --- | --- | --- |
| **Date** | **Paper** | **From** | **To** | **Subject** |
| 30/10/2017 | Holmes | Nicola Filardi | Sandra Holmes | Query as to provision of raw or t-scores in the paper. |
|  | Holmes | No reply received. | | |
| 30/10/2017 | Chapman, Robinson | Nicola Filardi | James Chapman | Request for normative data for two versions of the Perception of ability Scale for Students (1979 & 1992). |
| 30/10/2017 | Chapman, Robinson | James Chapman | Nicola Filardi | Provision of manual and data. |
| 23/11/2017 | Somerville | Nicola Filardi | David Leach | Request for standard deviations that were not included in paper. |
| 27/11/2017 | Somerville | David Leach | Nicola Filardi | Relevant material no longer accessible to the author. |
| 15/12/2018 | Gold | Nicola Filardi | John Johnson | Request for norms of Form C of the Coopersmith Inventory |
| 16/12/2018 | Gold | John Johnson | Nicola Filardi | John was not in possession of the norms. Referral to Lee Richmond who also worked on the study. |
| 16/12/2018 | Gold | Nicola Filardi | Lee Richmond | Request for norms of Form C of the Coopersmith Inventory |
| 18/12/2018 | Gold | Lee Richmond | Nicola Filardi | Lee was not in possession of the norms. |
