# Supplementary material for: Self-concept in poor readers: a systematic review and meta-analysis
Source: PeerJ. 2020 Mar 16;8:e8772. doi: 10.7717/peerj.8772 (PMC7081778; doi:10.7717/peerj.8772)
Supplement: Appendix S3 [file peerj-08-8772-s004.docx]

| **Appendix 3.** Risk of bias assessment form. Ratings (a-d) and stars (*) for each study are summarised in Table 2. | | | |
| --- | --- | --- | --- |
| **Authors:** | | **Date:** | |
| **Reviewer:** | | **Date:** | |
| **Sample size (maximum 4 points/stars)** | Representativeness | a) Representative of target population * |  |
|  |  | b) Somewhat representative of target population* |  |
|  |  | c) Selected group of users |  |
|  |  | d) No description of the sampling strategy |  |
|  | Sample size | a) Justified and satisfactory (i.e., sample large enough to detect small effects with power .8) * |  |
|  |  | b) Not justified |  |
|  | Response rate | a) Response rate satisfactory (i.e. > 60%) * |  |
|  |  | b) Response rate unsatisfactory (i.e., <60%) |  |
|  |  | c) No description of response rate |  |
|  | Reading assessment | a) Standardized reading assessment with data reported ** |  |
|  |  | b) Standardised reading assessment with no data reported |  |
| **Group comparability (maximum 2 points/stars)** | Groups comparable based on the study design or analysis. Confounding factors controlled | a) English poor readers * |  |
|  |  | b) Control for additional factors (attention, age, sex, SES, neurological or medical problem)* |  |
| **Self-concept assessment (maximum 2 points/stars)** | Assessment of outcome | a) Normed index of self-concept * |  |
|  |  | b) Questions presented and read out loud to the participants* |  |
|  | Statistical analysis | a) Statistical test clearly described and appropriate* |  |
|  |  | b) Statistical test is not appropriate, not described or incomplete |  |
| **Total:** |  |  |  |
| **Incomplete outcome data:** | | | |
| **Selective reporting:** | | | |
| **Other bias:** | | | |
