# Supplementary material for: Self-concept in poor readers: a systematic review and meta-analysis
Source: PeerJ. 2020 Mar 16;8:e8772. doi: 10.7717/peerj.8772 (PMC7081778; doi:10.7717/peerj.8772)
Supplement: Appendix S4 [file peerj-08-8772-s005.docx]

| **Appendix 4.** Studies excluded from review that did not meet inclusionary criteria | |
| --- | --- |
| **Study** | **Reason for exclusion** |
| Alexander-Passe (2006) | Definition of poor reading ability did not meet this review’s criteria |
| [Anderson (2009)](http://dx.doi.org/10.1111/j.1467-9604.2009.01400.x) | Case study only |
| [Anyanwu (2001)](https://doi.org/10.1515/IJAMH.2001.13.3.191) | Study did not include a standardised reading test (questionnaires only) Study did not include a standardised measurement of self-concept |
| [Arvans (2010)](https://search.proquest.com/docview/305031916/?pq-origsite=primo) | Study used measurement of comprehension to assess reading ability |
| [Athey (1982)](http://psycnet.apa.org/record/1983-10652-001) | Study was a review |
| Axelrod (1982) | Study did not include an entire set of reading data |
| [Baker (2007)](http://dx.doi.org/10.1016/j.ijlp.2007.09.010) | Study included unsuitable data for analysis (Q scores only) |
| [Baldridge (2011)](https://search.proquest.com/docview/858071083) | Study did not include an entire set of reading data |
| [Bull (2007)](https://doi.org/10.1016/j.ctcp.2006.07.003) | Definition of poor reading ability did not meet this review’s criteria. |
| [Burgess (1997)](https://ovidsp.tx.ovid.com/sp-3.31.1b/ovidweb.cgi?QS2=434f4e1a73d37e8c1cc07622d116f55143c9016f916daae7befdaa0d11c75c537583762bb0875063ec464894a564e5dc4db65ae66e7dd595f7a5477f0037117c2bff8c98363411f8238d6c2f5e65276c6e63fe148a51fdb0a3ef05fb489d2a29c4f64296eb1dd8c9bcf82b6924dcf848131e8774cedff278837aaf33f6463e857f01a74ebfb092327bf4cc9127ea24b8bd2ad07f75ee0db6dacf983234a243de10a8d5fc63aa00e4b67fbfda7be776f619f81a64d54c7a87f5169bfc5f9072cd1dd8c115225362d0005e19c4b5a2c5bd) | Study did not include a standardised reading test. |
| [Burton (2004)](https://doi.org/10.1080/0266736042000180410) | Study did not meet this review’s criteria for sample size (n = 7). Study did not include a standardised reading test. |
| [Busby (1974)](https://www.jstor.org/stable/20150940) | Correlational study with no clear group of participants with reading difficulties. |
| [Butkowsky (1980)](http://dx.doi.org/10.1037/0022-0663.72.3.408) | Study did not include a standardised measurement of self-concept. |
| [Callahan (2002)](http://ovidsp.ovid.com/ovidweb.cgi?T=JS&CSC=Y&NEWS=N&PAGE=fulltext&D=psyc4&AN=2002-95014-268) | Study included unsuitable data for analysis (unidentifiable format of scores). |
| [Carawan (2016)](https://doi.org/10.1080/13607863.2015.1008984) | Definition of poor reading ability did not meet this review’s criteria.  (self-identified dyslexia). |
| [Casey (1992)](https://www.ncbi.nlm.nih.gov/pubmed/1506463) | Study included unsuitable data for analysis (mean difference of 9 months only). |
| [Chapman (1997)](https://www.ncbi.nlm.nih.gov/pubmed/9376307) | Correlational study with no clear group of participants with reading difficulties. |
| [Chapman (2003)](https://doi.org/10.1002/dys.238) | Study was a review. |
| Cheatham (1968) | Definition of poor reading ability did not meet this review’s criteria. |
| [Chilton (2009)](https://ovidsp.tx.ovid.com/sp-3.31.1b/ovidweb.cgi?QS2=434f4e1a73d37e8c5a069e886e3737f243bcf8ea096469a809aba66ab5d20723544b85270248445dd069c1f08302c2b6724da87f22fcaddd3f1f25eb372788e2e5eafc17881a3725166164de5f3c77e7a7868d7cb5c68afa4a2befb16ed039bcec7dcf652ab8297aeb838a038ed8b9c71ff2c9f5b05094fde7d9ddc58e15df5788281a1961e2f6a589013e01f89d546875473d640c4b1e608f014535a435b2570fa171301f6bb4073bbeb36441bc3ede9ab543efefc0bf4c2bbe067a069ebc1e70831b441aa990ceeed65bbc16c2922a14845672befd5e654085ed4e4454b3700b1f0ab437ab0b2532e413c7663a93a39baf948048e150c6e6eb5b5312bcf03e) | Study used measurement of comprehension to assess reading ability. |
| [Chiu (2012)](https://doi.org/10.1177/0022219411431241) | Correlational study with no clear group of participants with reading difficulties. |
| [Collins (2001)](https://multisearch.mq.edu.au/primo-explore/fulldisplay?docid=TN_ericEJ624650&context=PC&vid=MQ&search_scope=PC_PLUS_LOCAL&tab=books_more&lang=en_US) | Study did not meet this review’s criteria for sample size. |
| [Cordoni (1979)](https://doi.org/10.1007/BF02653748) | This study did not report any data. |
| [Crochet (1999)](https://ovidsp.tx.ovid.com/sp-3.31.1b/ovidweb.cgi?QS2=434f4e1a73d37e8c40659e682b880c52cfc58b3c3f2d641297c9954e1fba332bfe2f1aea8006443781106ad3f7e75ec8e0b1d94a91321a29feed0853651a9ff1a400a5d843b8221ac591813151a57241dd881b3a4d91badc55afd5cc8749359da0ec997848cd6bd24d45069e433802514a0d7b5b7e3a4c70823a41bf15fad3164695c0bed8ced19d377123fc39e1aa3047597ae28e1798d55d2fd817c3bdbb3cf1608d46188e4d4585af526cce9279ba288b141f13bee0c8be7207af9a34d9bc5186b6052f5fbefa9b30b51cd398dead) | Case study only. |
| [Daki (2010)](http://dx.doi.org/10.1080/00220670903383127) | Participants had comorbidities which did not meet this review’s criteria. |
| Davisson (1979) | Definition of poor reading ability did not meet this review’s criteria. |
| [Desnouee (2001)](http://ovidsp.ovid.com/ovidweb.cgi?T=JS&CSC=Y&NEWS=N&PAGE=fulltext&D=psyc3&AN=2001-95001-174) | Study used measurement of comprehension to assess reading ability. |
| [Elkind (1996)](http://dx.doi.org/10.1007/BF02648175) | Study did not include a standardised measurement of self-concept. |
| [Ellis (1996)](https://ovidsp.tx.ovid.com/sp-3.31.1b/ovidweb.cgi?QS2=434f4e1a73d37e8c79e5d8c142641a544d3b3e4225e43d7c60db15dbd95371a274f6edcb49a9136c5523773ba637ff99e4399f72dbc37f6fb2bc9ae67253c501381062afcba8e648d536cbb6496d491e4e9bc02a7c3cbf916cd0a99fa6215886dd85f1962cd50a232eff8f8cb69be2419ce7b331a8c4f325ec54ea7c531fa7f18723f452729896f61063d796f82f25bdc0e9b52768eed0ccf98be0d082e2cd257e8b0c0c6c3ffed4629d15238b95550cbc160295670d7499b2d59cab8b25bfdb1f6abafd97b1f4b65fed0e5f53f933d7) | Study did not include a standardised reading test. |
| [Enns (2007)](http://dx.doi.org/10.1353/aad.2007.0011) | Case study only. |
| [Feiwell (1997)](http://ovidsp.ovid.com/ovidweb.cgi?T=JS&CSC=Y&NEWS=N&PAGE=fulltext&D=psyc3&AN=1997-95017-154) | Study did not include a standardised reading test. |
| [Fisher (1986)](https://ovidsp.tx.ovid.com/sp-3.31.1b/ovidweb.cgi?QS2=434f4e1a73d37e8c9c8be07760ee3a7a897c4b632b3bf14035558846b3307b7b8e093b9d17062bc0646ac2ac4480ea28add1541c4627b379fb47bccb15826021cba7be6c21c2fa3525dfad3a4ed9e24ef3fdad9c5c94894a2f0a36c5ae4ab1e62d9bb8deab67b8fdca022e12e9749fd29a59bcf35acdec28ec2d268fb817bcb31aa187716dd0afc31ed92797925b9a9f4c301d7880a21f9f7c0a16a85498bc4d5d4b0f584c95e529efa2961e8c2be285ca2cb05bed14f8747408355a7ce35dd0adfde98a976c12ae9bb781f2509abef6f27793dfe0d713be) | Study included unsuitable data for analysis (scores were not standardised). |
| [Freeze (2005)](https://ovidsp.tx.ovid.com/sp-3.31.1b/ovidweb.cgi?QS2=434f4e1a73d37e8ccc0b611cd6b5b34de304ac589b575267b7f6674c2a0322fa5d48f2cac07cbab01134fe774563762d272aedfb031e874d19e6e1c2bb74e26ead382decef9d8c487600fff22cd807a6858bd730133171b9e0906a74b5625362886dec4fec89b18a32b7d8af35e6014664943b29e1e4481d6cd3bd4096e011a2c37a25143d39ba096c453c6f19b946ae365a059491f973b1696b4cf3b5c3a20aac3946be2ca539d019f8edc2872c477da393ce3987c312900458ab40ecba97419b82408de6c83b2edee67d8f3776e32261a21852425c17dd) | Definition of poor reading ability did not meet this review’s criteria. |
| [Gentry (1995)](https://doi.org/10.1177/08830738950100S121) | Case study only. |
| [Gickling (1974)](https://www.ncbi.nlm.nih.gov/pubmed/4412552) | Definition of poor reading ability did not meet this review’s criteria. |
| [Glazzard (2010)](https://doi.org/10.1111/j.1467-9604.2010.01442.x) | Study did not meet this review’s criteria for sample size (N=9). Study did not include a standardised reading test. |
| [Glick (1972)](https://ovidsp.tx.ovid.com/sp-3.31.1b/ovidweb.cgi?WebLinkFrameset=1&S=AIGMFPJIGLDDEAMNNCEKNBDCINAJAA00&returnUrl=ovidweb.cgi%3fMain%2bSearch%2bPage%3d1%26S%3dAIGMFPJIGLDDEAMNNCEKNBDCINAJAA00&directlink=https%3a%2f%2fovidsp.tx.ovid.com%2fovftpdfs%2fFPDDNCDCNBMNGL00%2ffs046%2fovft%2flive%2fgv023%2f00004760%2f00004760-197206000-00011.pdf&filename=Some+social-emotional+consequences+of+early+inadequate+acquisition+of+reading+skills.&navigation_links=NavLinks.S.sh.138.1&link_from=S.sh.138%7c1&pdf_key=FPDDNCDCNBMNGL00&pdf_index=/fs046/ovft/live/gv023/00004760/00004760-197206000-00011&D=psyc2&link_set=S.sh.138%7C1%7Csl_10%7CresultSet%7CS.sh.138.139%7C0) | Definition of poor reading ability did not meet this review’s criteria. |
| Griffiths (1970) | Study did not include an entire set of reading data. Study did not include a standardised measurement of self-concept. |
| [Hanich (2004)](http://dx.doi.org/10.3200/JOER.97.5.227-234) | Definition of poor reading ability did not meet this review’s criteria. |
| [Holopainen (2017)](https://doi.org/10.1080/1034912X.2016.1181257) | Participants in this study were not native English speakers (Finland). |
| [Howarth (2001)](https://search.proquest.com/docview/851539869) | Study did not include a standardised measurement of self-concept. |
| [Humphrey (2002)](https://doi.org/10.1111/1467-8527.00234) | Study did not include a standardised reading test. |
| [Hunter (1971)](http://dx.doi.org/10.1177/002221947100401006) | Self-concept measurement did not match this review’s criteria for assessment (Good-Enough Harris Draw-A-Man Test). |
| [Husak (1979)](https://doi.org/10.2466/pms.1979.48.2.447) | Correlational study with no clear group of participants with reading difficulties. |
| [Jensen (2000)](https://doi.org/10.1207/S15324826AN0704_4) | Participants in this study were not native English speakers (Sweden). |
| [Jordan (2014)](https://doi.org/10.1002/dys.1478) | Study included unsuitable data for analysis. |
| Kenny (1982) | Definition of poor reading ability did not meet this review’s criteria. |
| [Lawrence (1985)](https://doi.org/10.1080/0013188850270306) | Study included unsuitable data for analysis (change scores only). |
| [Levinson (2003)](http://ovidsp.ovid.com/ovidweb.cgi?T=JS&CSC=Y&NEWS=N&PAGE=fulltext&D=psyc4&AN=2003-95022-182) | Study did not include a standardised reading test. |
| [Lewis (1984)](https://www.jstor.org/stable/23901081) | Reading assessment did not match this review’s criteria for the measurement of poor reading. |
| [Macphail (2013)](http://gateway.proquest.com/openurl?url_ver=Z39.88-2004&rft_val_fmt=info:ofi/fmt:kev:mtx:dissertation&res_dat=xri:pqm&rft_dat=xri:pqdiss:3523358) | Study used measurement of comprehension to assess reading ability. |
| Marble (1974) | Definition of poor reading ability did not meet this review’s criteria. |
| [Martin (1977)](http://dx.doi.org/10.1177/002221947701000503) | Case study only. |
| Maughan (1995) | Study was a review. |
| [Maughan (1996)](http://dx.doi.org/10.1111/j.1469-7610.1996.tb01421.x) | Definition of poor reading ability did not meet this review’s criteria.  (longitudinal follow-up study where adults had recovered). |
| McClain (1971) | Study used measurement of comprehension to assess reading ability. |
| McGrath (1977) | Definition of poor reading ability did not meet this review’s criteria. |
| [Meyer (2002)](http://dx.doi.org/10.1207/S15326985EP3702_5) | Study did not include an entire set of reading data. |
| [Migden (1990)](http://dx.doi.org/10.1007/BF02648143) | Case study only. |
| [Milani (2010)](https://doi.org/10.1002/dys.397) | Participants in this study were not native English speakers (Italy). |
| [Miller (2010)](https://doi.org/10.1348/000709909X481652) | Definition of poor reading ability did not meet this review’s criteria. |
| [Nalavany (2012)](https://doi.org/10.1002/dys.1433) | Definition of poor reading ability did not meet this review’s criteria  (self-identified dyslexia). |
| [Nalavany (2015)](http://dx.doi.org/10.1093/bjsw/bct152) | Study did not include a standardised reading test. |
| [Nicholls (1979)](http://dx.doi.org/10.1037/0022-0663.71.1.94) | Definition of poor reading ability did not meet this review’s criteria. |
| [Novita (2016)](https://doi.org/10.1080/08856257.2015.1125694) | Definition of poor reading ability did not meet this review’s criteria. |
| [Novosel (2013)](https://search.proquest.com/docview/1038153874) | Sample was too varied in disabilities and comorbidities to match this review’s criteria. |
| Oberman (1981) | Definition of poor reading ability did not meet this review’s criteria. |
| [Pisecco (1997)](http://ovidsp.ovid.com/ovidweb.cgi?T=JS&CSC=Y&NEWS=N&PAGE=fulltext&D=psyc3&AN=1997-95001-158) | Study included unsuitable data for analysis (mean scores without standard deviations). |
| [Polychroni (2006)](http://dx.doi.org/10.1080/08856250600956311) | Participants in this study were not native English speakers (Greece). |
| [Prochnow (2013)](http://dx.doi.org/10.1080/1034912X.2013.812188) | This study did not match this review’s criteria for the definition of the poor reading group. |
| [Rapp (1992)](http://ovidsp.ovid.com/ovidweb.cgi?T=JS&CSC=Y&NEWS=N&PAGE=fulltext&D=psyc3&AN=1992-77621-001) | This study did not include self-esteem data. |
| [Retelsdorf (2014)](http://dx.doi.org/10.1016/j.learninstruc.2013.07.004) | Participants in this study were not native English speakers (Germany). |
| [Reynolds (2000)](https://www.ncbi.nlm.nih.gov/pubmed/17557685) | Definition of poor reading ability did not meet this review’s criteria.  Study did not include a standardised measurement of self-concept. |
| [Riddick (1999)](http://dx.doi.org/10.1002/(SICI)1099-0909(199912)5:4%3c227::AID-DYS146%3e3.0.CO;2-6) | This study did not include a measurement of reading ability. |
| [Robeck (1964)](https://doi.org/10.2466/pms.1964.19.1.7) | Study did not include a standardised reading test. |
| [Roberts (2016)](https://escholarship.org/uc/item/4m38599n) | Definition of poor reading ability did not meet this review’s criteria. |
| Rodgers (2017) | Study did not include a standardised reading test. |
| [Rosenthal (1973)](https://doi.org/10.1177/105345127300900104) | Definition of poor reading ability did not meet this review’s criteria. |
| [Sadovnik (1999)](https://search.proquest.com/docview/304668536?pq-origsite=primo) | Study was a review. |
| [Scanlon (2014)](http://dx.doi.org/10.1037/h0100577) | This study did not include a measurement of reading ability. |
| [Smith-Spark (2016)](https://doi.org/10.1002/dys.1528) | Self-concept measurement did not match this review’s criteria for assessment (questionnaires regarding memory). |
| [Snowling (2007)](https://doi.org/10.1111/j.1469-7610.2006.01725.x) | Definition of poor reading ability did not meet this review’s criteria. |
| [Somerville (1998)](https://doi.org/10.1080/0013188880300106) | Study included unsuitable data for analysis (standard deviations unable to be located by the authors). |
| [Svensson (2001)](https://doi.org/10.1002/dys.178) | Participants in this study were not native English speakers (Sweden). Study did not include a standardised measurement of self-concept. |
| [Svensson (2003)](http://dx.doi.org/10.1023/A:1025832815286) | Participants in this study were not native English speakers (Sweden). |
| [Svennson (2014)](https://doi.org/10.1023/A:1025832815286) | Participants in this study were not native English speakers (Sweden). |
| Tam (2012) | This finding was a poster. A published article could not be located. |
| [Terras (2009)](https://doi.org/10.1002/dys.386) | Study did not include an entire set of reading data (mean and standard deviation). |
| [Thomas (2016)](https://doi.org/10.1016/j.jaac.2016.09.157) | This finding was a poster. A published article could not be located. |
| [Thomson (1980)](http://psycnet.apa.org/record/1981-28278-001) | Study did not include a standardised reading test. |
| [Undheim (2008)](https://doi.org/10.1111/j.1467-9450.2008.00661.x) | Participants in this study were not native English speakers (Norway). |
| [van Kraayenoord (1999)](http://dx.doi.org/10.1007/BF03173117) | Participants in this study were not native English speakers (Germany). |
| [van Kraayenoord (2003)](http://dx.doi.org/10.1007/BF03173605) | Participants in this study were not native English speakers (Germany). |
| Wattenberg (1964) | Definition of poor reading ability did not meet this review’s criteria. |
